# Hybrid endovascular and surgical staged approach for mycotic carotid pseudoaneurysms: a case report and literature review

**DOI:** 10.3389/fsurg.2024.1394441

**Published:** 2024-07-09

**Authors:** Sallustro Marianna, Ficarelli Ilaria, Prisco Teresa, Pontarelli Armando, Foschini Giuseppe, Toni Marisole, Rinaldi Valerio, Nardi Priscilla, Piergaspare Palumbo, Illuminati Giulio, D’Andrea Vito, Ruotolo Carlo

**Affiliations:** ^1^ Vascular Surgery Unit, Cardarelli Hospital, Naples, Italy; ^2^ Department of Surgical Sciences, University of Rome “La Sapeinza”, Rome, Italy

**Keywords:** mycotic, carotid, pseudoaneurysm, graft, stentgraft, vein, extracranial, hybrid

## Abstract

**Background:**

Mycotic carotid pseudoaneurysms represent a challenge for surgeons. They are rare and associated with high mortality and morbidity.

**Methods:**

We reported a case of a 61-year-old man with a mycotic pseudoaneurysm of carotid bifurcation. The case was managed by a staged procedure, starting with initial endovascular control using a stent graft, followed by open arterial reconstruction using a saphenous vein graft.

**Results:**

The patient was discharged home with a patent carotid artery and no sign of infection or bleeding. A computed tomography scan performed at 1 month, 6 months, and 1 year later confirmed good patency of the graft without imaging of cerebral ischemia.

**Conclusions:**

Mycotic pseudoaneurysms of the extracranial carotid artery are rare and should always be treated surgically. This disease, despite its rarity, requires early detection and treatment to avoid fatal outcomes. A hybrid staged approach is suggested, compared to one-staged surgery, to avoid rupture and improve clinical outcomes. This approach involves using a stent graft combined with antibiotic therapy as bridge treatment until definitive surgery can be performed to enable arterial reconstruction with an autologous graft.

## Introduction

Mycotic aneurysms of the extracranial carotid artery are relatively rare, with only a small number of cases reported in the literature (1, 2); despite their rarity, they pose a significant threat to patient health and wellbeing. These aneurysms are associated with high rates of mortality and morbidity (3) and can lead to serious complications such as rupture and metastatic brain abscesses (1).

The clinical presentation of these aneurysms is characterized by a growing and pulsatile cervical lump, often accompanied by pain, tenderness, fever, hoarseness of voice (dysphonia), and difficulty swallowing (dysphagia) (4, 5). The condition is characterized by unspecific symptoms making preoperative diagnosis very challenging, especially when no risk factors are reported (6). A definitive diagnosis hinges on identifying a microbial pathogen within a tissue sample, which is typically bacterial (5), despite the term “mycotic” implying a fungal origin (6). The treatment of these patients represents a challenge for surgeons. The preferred treatment option is the surgical removal of the aneurysm, followed by the reconstruction of the damaged artery, typically using an autologous conduit in a one-staged approach. The use of stent grafts can be useful to avoid the imminent risk of pseudoaneurysm rupture; however, an exclusively endovascular approach to treating infected aneurysms is a subject of ongoing debate, given limited research on their long-term efficacy (7). A hybrid endovascular and surgical staged repair initially gives an opportunity to successfully manage the acute pseudoaneurysm with endovascular covered stents. This approach also allows the clinicians to administer some days of antibiotic therapy, thereby reducing the surgical risk and improving the surgical outcome by preventing delayed complications (8, 9).

The aim of our case report is to describe the successful treatment of a mycotic pseudoaneurysm of carotid bifurcation with a staged hybrid approach and review the specific existing literature. According to our experience, a multistep hybrid procedure, using an endovascular approach for initial control followed by definitive surgery, should be considered useful and effective for this rare pathology compared to one-staged surgery, especially in an emergency setting.

## Methods

Since this case report utilized anonymized data, informed consent was not necessary.

In February 2024, an online search was conducted in the Medline and Embase databases using specific MeSH terminology (Carotid Mycotic Pseudoaneurysm, Aneurysm, Adult).

## Case report

**Figure 1:**
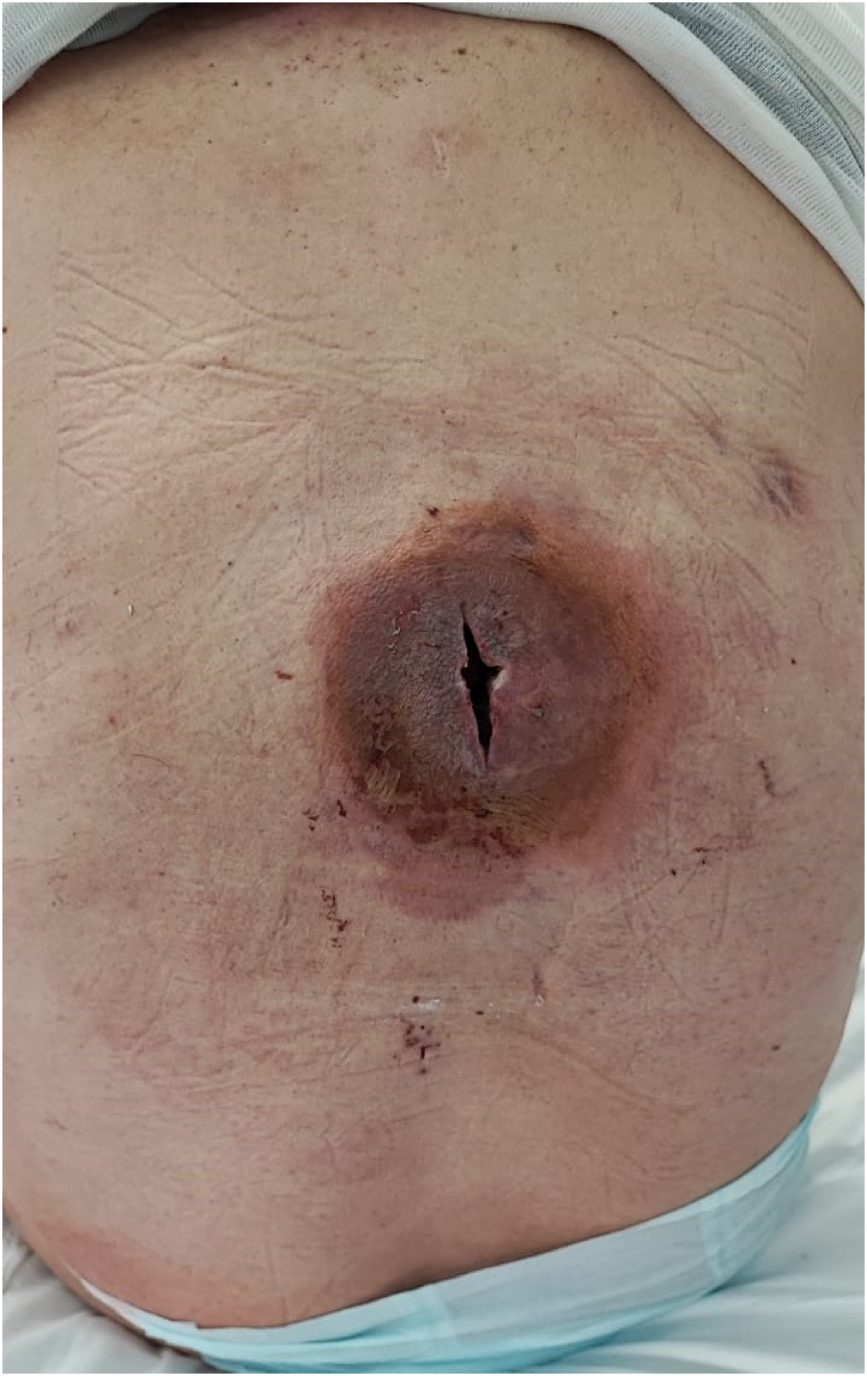
Huge dorsal abscess opened and drained with the microbiological examination positive for *Staphylococcus aureus*.

**Figure 2:**
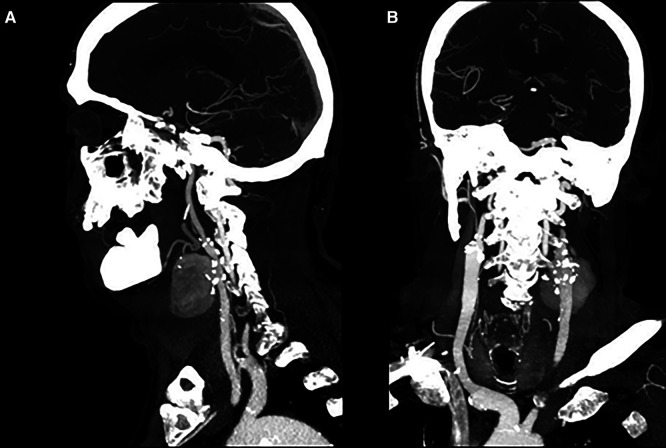
(**A,B**) CT scan in LL and AP projection showing a 48-mm × 38-mm pseudoaneurysm of the left carotid bifurcation without any cerebral embolic signs. LL, laterolateral; AP, anteroposterior.

**Figure 3:**
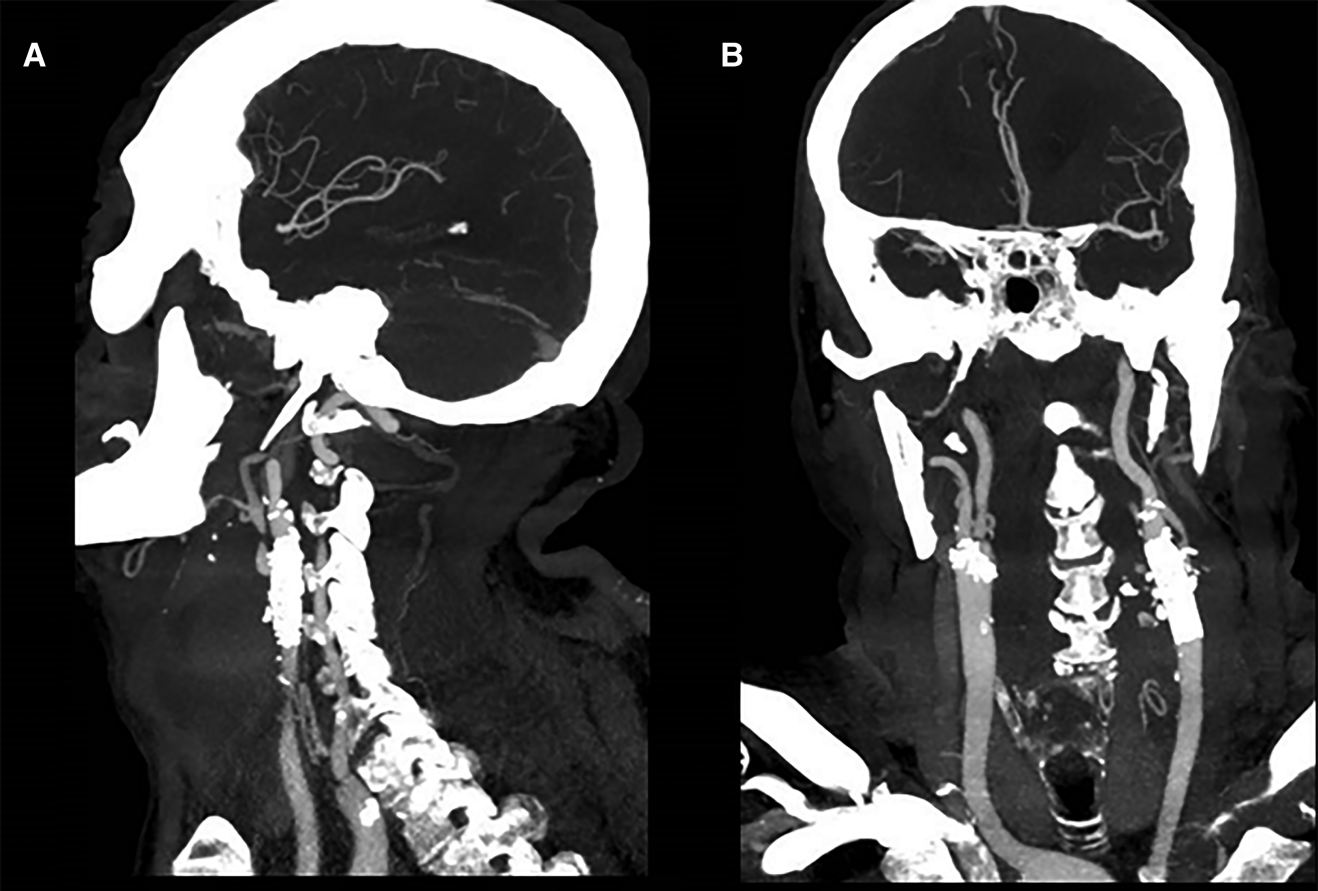
(**A,B**) CT control in AP and LL projection after endovascular exclusion of the mycotic pseudoaneurysm using a stent graft. LL, laterolateral; AP, anteroposterior.

A 61-year-old Caucasian male was admitted to the Emergency Care unit of our hospital after left cervical swelling appeared for the last 10 days without dysphagia or neurological deficits. He had a history of hypertension, smoking addiction (now ceased), diabetes, and atrial fibrillation. The patient denied any prior neck or oral surgery. Vital signs were stable, with a normal body temperature of 36°C. Physical examination revealed a pulsatile, firm, and painless mass in the left anterior neck, just above the clavicle. The overlying skin appeared normal and not reddened. No neurological focal signs of central or peripheral origin were present. However, there was a huge dorsal abscess, which was immediately opened and drained (Figure 1). The abscess wall was submitted for culture and tested positive for *Staphylococcus aureus*. Blood tests demonstrated leukocytosis with a white blood cell count of 22.53 × 10^3^/μl. Transthoracic echocardiogram was negative for endocarditis. Based on clinical findings and imaging studies, our diagnosis was a mycotic pseudoaneurysm (Figures 2A, B). Following the recommendation of our infectious consultant, the patient was promptly initiated on antibiotic therapy with Tazocin (Pfizer, Italy) die 4.5 mg thrice; serial hemocultures were made but were negative. Surgical resection and revascularization with autologous material were mandatory. To reduce the surgical risk, the patient was first sent to radiology. He was taken to the interventional radiology suite for an endovascular exclusion using an 8 mm × 38 mm Advantia (Atrium medical corporation, Gentinge Australia Pty Ltd) balloon-expandable covered stent that was deployed across the healthy segment of the common carotid artery (CCA) proximally and the internal carotid artery (ICA) distally (Figures 3A,B), without embolization of the external carotid artery. A loading dose of aspirin and clopidogrel was administrated before the deployment of the stent graft.

**Figure 4:**
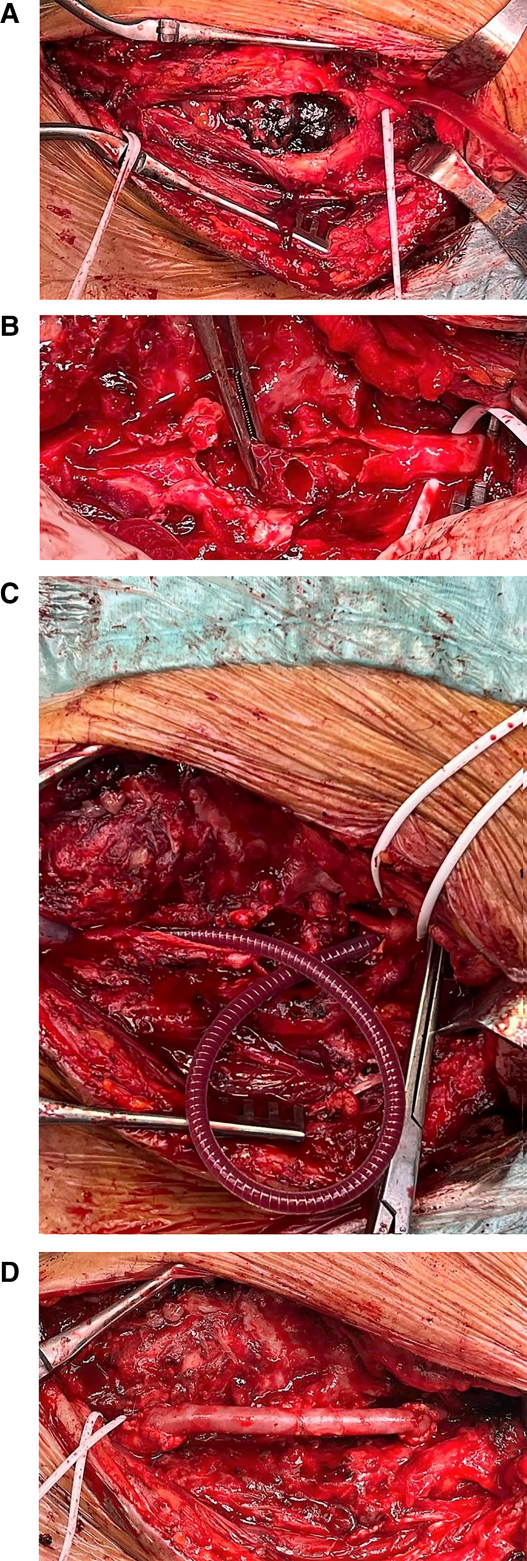
(**A**) Intraoperative image showing the pseudoaneurysm sac opened. (**B**) Intraoperative image showing stent graft removal. (**C**) Intraoperative image showing the Sundt shunt for cerebral perfusion. (**D**) Open surgical carotid artery reconstruction using the great saphenous vein graft.

**Figure 5:**
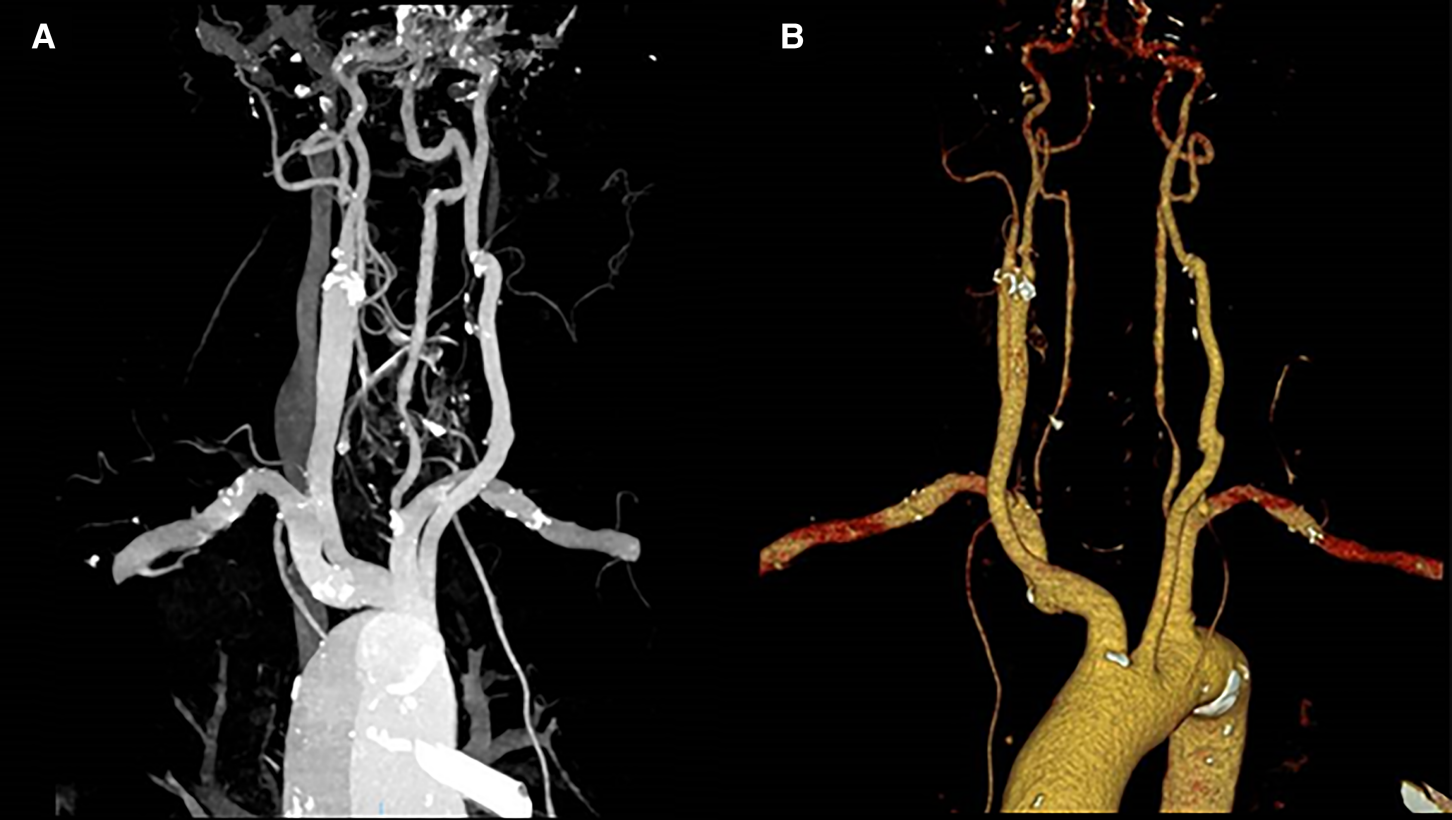
(**A**,**B**) 1-year CT scan showing good patency of the graft without imaging of cerebral ischemia.

One week later, the patient received general anesthesia for the procedure. Cerebral perfusion was monitored by near-infrared spectroscopy (NIRS) and maintained using a Sundt shunt. A standard surgical approach was employed, involving an incision along the anterior border of the sternocleidomastoid muscle. We achieved control of internal, external, and common carotid arteries and dissected the pseudoaneurysm. Then, we clamped, respectively, the common, internal, and external carotid arteries. The pseudoaneurysm sac was opened, the previously placed stent graft was extracted, and the surrounding inflammatory tissue was debrided (Figure 4A). A specimen of the pseudoaneurysm wall was obtained and submitted for microbiological analysis (positive for *S. aureus*). The external carotid artery was ligated. After obtaining proximal and distal control, a large portion of the infected carotid artery, including the stent, was surgically removed, and cerebral perfusion was maintained using a Sundt shunt (Figures 4B,C). The shunt was initially inserted into the proximal and distal stumps of the carotid artery and then through the venous graft after the first anastomosis. The resected segment was replaced with a similarly sized section of the great saphenous vein (Figure 4D). The postoperative period was uneventful, with no neurological complications. Following antibiotic therapy with teicoplanin and doxycycline, the patient's white blood cell count and all inflammatory markers normalized; he was discharged after 12 days from surgery and continued antibiotic therapy (doxycycline 100 mg twice daily) for another 3 weeks, with daily aspirin 100 mg prescribed on a lifelong schedule. Follow-up computed tomography (CT) scans at 1 month, 6 months, and 1 year demonstrated patency of the graft (Figures 5A,B) with no signs of cerebral ischemia.

## Discussion

Although uncommon, mycotic extracranial carotid pseudoaneurysms can develop as a complication of local or systemic infections, including dental suppuration, bacterial sinusitis, Lemierre's syndrome, bacterial endocarditis, and bacteremia (7, 10). A primary infection can cause a carotid pseudoaneurysm and then lead to perforation and an abscess. However, some researchers propose that the abscess might be the initial lesion that subsequently breaches the arterial wall. In addition, other local inflammatory conditions affecting blood vessels, such as vasculitis or hereditary dysfunctions, could potentially weaken the arterial wall and increase susceptibility to infection (11). Although trauma is the most frequent cause of aneurysms in general (42%), the source of infection in mycotic aneurysms often remains unidentified (25%) (12). In our patient's case, despite good overall health aside from the dorsal abscess, the specific origin of the infection remained unclear. The most common bacterial pathogens associated with mycotic carotid aneurysms are *Staphylococcus*, *Streptococcus*, and *Salmonella* (4).

Nowadays, the primary treatment for mycotic pseudoaneurysms involves a combination of long-term, broad-spectrum systemic antibiotics to eradicate the infection and surgical intervention to remove the infected tissue and reconstruct the affected blood vessel. Surgical management typically involves excision of the pseudoaneurysm, debridement of the infected tissues, and restoration of arterial continuity using different techniques such as primary closure, patch angioplasty, bypass, and resection with primary anastomosis, which can lead to complications such as thrombosis, embolization, and rupture. Although various graft materials such as autologous veins, synthetic options such as prostheses impregnated with silver salts, and cryopreserved allografts exist for reconstruction, the potential for long-term complications due to infection must be considered. These complications can include stenosis, a narrowing of the graft, or dilatation, an abnormal widening. Autologous tissues such as the superficial femoral artery, the hypogastric artery, and the saphenous vein are currently considered the preferred material for vascular reconstruction because of their resistance to infection and their immediate availability (13–15).

Open surgery in an acute setting is generally associated with poor outcomes, including stroke and mortality of up to 50% of patients if the carotid artery is ligated. This intervention carries a mortality risk of 25%–60% with a high incidence of stroke, so it is suggested that this procedure be performed with an intact circle of Willis. In emergencies, a synthetic prosthesis is often used to reconstruct the carotid artery because there is not enough time to evaluate and prepare a vein graft, and cryopreserved arterial allografts are not available in the institute (16). Endovascular repair using a covered stent has been described as a “bridge” solution, often in emergent settings, before early definitive surgical management (17–19), or in high-risk patients who are not candidates for open surgery (7). Endovascular repair using coils offers a valuable temporary alternative for treating patients with pseudoaneurysms presenting to emergency care with carotid blowout syndrome (20, 21). However, its usefulness is limited by the potential risks of placing prosthetic material (coils) in an already infected area. Another treatment option is the sacrifice of the carotid axis through embolization with coils, preceded by an angiographic occlusion test to verify intracranial compensation.

In our situation, to mitigate the risk of septic emboli and rupture during surgery, we opted for administering endovascular bridge therapy to the patient. This involved utilizing a covered stent graft in conjunction with antibiotic treatment prior to proceeding with the surgical intervention until a reduction in the volume of the mass in the neck was evident.

However, the major advantage of using a covered stent as bridge therapy is the administration of antibiotic therapy for a sufficient time before definitive surgery. Antimicrobial drugs strongly improve clinical outcomes, minimizing the risk of hemorrhagic and ischemic complications (i.e., septic emboli) originating from the pseudoaneurysm while waiting for surgery.

We hold the opinion that employing endovascular stent graft placement as the primary treatment approach is optimal for preventing rupture and managing hemorrhage in patients diagnosed with carotid blowout syndrome. However, in cases where patients have realistic prospects for long-term survival, reconstruction should also be deliberated upon (19). In our view, stent placement should be succeeded by definitive reconstruction to minimize both the mortality and morbidity risks associated with thrombosis, re-bleeding, infection, and the necessity for carotid ligation or embolization. The hybrid treatment of mycotic carotid pseudoaneurysms has been scarcely reported in the literature, with follow-up limited to mid-term. We performed a literature review on mycotic common and internal carotid aneurysms, which revealed 18 papers reporting successful surgical treatment in adult patients for this disease (Table 1). Eighteen patients (16 males, 2 females; mean age = 64.9 years old) were treated for mycotic carotid pseudoaneurysms—carotid bifurcation (50%), CCA (39%), and ICA (11%). The preferred approach was surgical treatment in 83% of the cases, and aneurysms were excluded by a surgical bypass in 61% of the cases using prevalently saphenous vein graft (55%), end-to-end anastomosis in 11%, and patch in the latter 11%. An endovascular approach was used in 12% of the cases (one case involving embolization and one involving endovascular repair using a covered stent). A hybrid staged approach was described only once in the literature for a 79-year-old man with a mycotic pseudoaneurysm involving carotid bifurcation that developed after a dental extraction. The reported technical success rate (TS) was 100%. The mean of postoperative follow-up, considering the time available in the literature and not reported for all the cases, was 6.7 months (range 1–12 months). This revealed vessel patency in 12 patients; for six patients, the follow-up result was not reported. Postoperative complications were fundamentally neurological (cordal palsy and recurrent, hypoglossal, and facial palsy). Our case confirms that carotid bifurcation is the most frequent site involved in pseudoaneurysm degeneration of the extracranial carotid axis, that patients usually present in stable hemodynamic condition, and that the hybrid staged approach represents a valid option for treating mycotic extracranial carotid pseudoaneurysms. At present, surgical management of a mycotic pseudoaneurysm invariably involves prolonged antibiotic treatment, initiated preoperatively and guided by culture results (5). Following surgery, antibiotic therapy is typically recommended for a minimum of 6 weeks, with certain authors advocating for continuation up to 6 months.

**Table 1:** Mycotic extracranial common or internal carotid pseudoaneurysm in the survived adult patient using full-text English articles.

	Age	Gender	Localization	Etiology	Intervention	Anesthesia	Shunt	Graft	Intraoperative bacteria	Antibiotic therapy type/duration	Postoperative complications	Follow-up (months)
Bordet et al. (10)	74	M	Carotid bulb	Lemierre's syndrome	Surgical bypass	General	No	Cryopreserved arterial allograft	*Fusobacterium necrophorum*	Amoxicillin clavulanate + metronidazole/6 weeks	No	Patent (6)
Deiana et al. (22)	54	M	Carotid bifurcation	Unknown	Surgical bypass	General	No	Saphenous vein	*Staphylococcus epidermidis*	Imipenem + teicoplanin/2 weeks Amoxicillin clavulanate/2 weeks	No	Patent (1)
Baranoski et al. (23)	51	M	Common carotid artery	Pharyngeal-carotid fistula after radiotherapy for larynx carcinoma	Surgical bypass	General	No	Superficial femoral artery	Not available	Not available	No	Patent (3)
Molina et al. (24)	68	M	Common carotid artery and bulb	Unknown (previous neck surgery for tumor)	Surgical bypass	General	No	Saphenous vein	*Staphylococcus aureus*	Vancomycin	No	Patent (12)
Kenyon et al. (25)	62	M	Common carotid artery	Unknown	Surgical bypass	NA	NA	Saphenous vein	Absent	NA	Complete cordal palsy	Patent (NA)
Benedetto et al. (26)	75	M	Common + internal + external carotid artery	Lemierre's syndrome	Surgical bypass	General	Yes	Saphenous vein	*Fusobacterium nucleatum*	Piperacillin/tazobactam + metronidazole Clindamycin/4 weeks	No	Patent (2)
Lui et al. (27)	50	M	Internal carotid artery	Tuberculosis	End-to-end anastomosis	General	No	None	*Mycobacterium tuberculosis*	Rifampicin + isoniazid + ethambutol/NA	First-bite syndrome	Patent (12)
Pirvu et al. (1)	56	M	Common + internal carotid artery	Unknown	Surgical bypass	NA	Yes	Saphenous vein	*Salmonella enteritidis*	Not specified/6 weeks	Recurrent nerve paralysis	Patent (6)
Wales et al. (17)	79	M	Carotid bifurcation	Dental extraction	Staged hybrid approach (covered stent + surgical bypass)	General	Yes	Saphenous vein	*Staphylococcus epidermidis*, *Staphylococcus capitis*, and *Proprionibacterium*	Not specified/6 weeks	Horner's syndrome + hypoglossal palsy	Patent (NA)
Bagia and Hall (3)	55	M	Common carotid artery	Septicemia following urinary tract infection	End-to-end anastomosis	NA	NA	None	*Klebsiella pneumoniae*	Ciprofloxacin/6 weeks	No	Patent
Raso et al. (28)	51	M	Common + internal carotid artery	Septicemia following carotid artery stenting (CAS)	Surgical bypass	NA	NA	Saphenous vein	*Staphylococcus aureus*	Oxacillin + linezolid + cefepime/8 weeks	No	NA
Kim et al. (29)	89	M	CCA	NA	Surgical bypass	NA	NA	Rifampicin-soaked polytetrafluoroethylene (PTFE)	*Staphylococcus aureus*	Ciprofloxacin/3 months	No	NA
Masumoto et al. (30)	56	F	CCA	Cervical abscess	Patch	NA	NA	Autologous pericardial patch	*Staphylococcus aureus*	Ampicillin + sulbactam	No	NA
Kaviani et al. (31)	78	M	Carotid bifurcation	Carotid-cutaneous fistula in irradiated neck	Surgical bypass	General	NA	Saphenous vein	*Staphylococcus aureus*	NA	No	NA
Grazziotin et al. (32)	49	M	ICA	Submandibular abscess	Patch	General	Yes	Saphenous vein	*Staphylococcus aureus*	Nafcillin + rifampin/6 weeks Levofloxacin/4 weeks	No	NA
Kasangana et al. (2)	55	F	CCA	Tuberculosis	Surgical bypass	General	No	Saphenous vein	*Mycobacterium tuberculosis*	Antituberculosis regimen/6 months	Transient facial fullness	Patent (12)
Mazzaccaro et al. (18)	81	M	CCA + ICA	Bacteremia	Endovascular repair	General	No	Covered stent	*Staphylococcus aureus*	Teicoplanin + ceftriaxone/4 weeks	No	Patent (7)
Amano et al. (33)	85	M	CCA	Vernet's syndrome	Embolization	NA	No	Coils	NA	NA	No	NA

## Conclusions

Mycotic pseudoaneurysms of the extracranial carotid artery are rare but present with a wide spectrum of symptoms. This disease, despite its rarity, requires early detection and treatment to avoid fatal outcomes. Frequently, the precise origin of the infection remains elusive. Treatment involves aneurysmectomy combined with antibiotic therapy, with the preferred approach being the restoration of arterial continuity using an autologous graft.

The utilization of a stent graft as a definitive solution remains controversial but is suggested as bridge therapy before definitive surgical treatment in urgent settings to avoid rupture, giving the possibility to administer preoperative antibiotic therapy for a sufficient period, which strongly improves clinical outcomes by minimizing the risk of hemorrhagic and ischemic complications.

A hybrid staged approach is useful and effective compared to one-staged surgery to avoid rupture and improve clinical outcomes using a stent graft and antibiotic therapy before definitive surgery and then achieve arterial reconstruction with an autologous graft.

## Data Availability

The original contributions presented in the study are included in the article/Supplementary Material, further inquiries can be directed to the corresponding authors.
